# Mental health impact of the COVID-19 pandemic in U.S. military veterans: a population-based, prospective cohort study

**DOI:** 10.1017/S0033291721002361

**Published:** 2023-02

**Authors:** Melanie L. Hill, Brandon Nichter, Peter J. Na, Sonya B. Norman, Leslie A. Morland, John H. Krystal, Robert H. Pietrzak

**Affiliations:** 1 Department of Psychiatry, University of California, San Diego, CA, USA; 2 VA San Diego Healthcare System, San Diego, CA, USA; 3 Department of Psychiatry, Yale School of Medicine, New Haven, CT, USA; 4 National Center for PTSD, White River Junction, VT, USA; 5 VA Center of Excellence for Stress and Mental Health, San Diego, CA, USA; 6 National Center for PTSD, Pacific Islands Division, Honolulu, HI, USA; 7 National Center for PTSD, VA Connecticut Healthcare System, West Haven, CT, USA; 8 Department of Social and Behavioral Sciences, Yale School of Public Health, New Haven, CT, USA

**Keywords:** Anxiety, COVID-19, depression, PTSD, psychiatric distress, veteran

## Abstract

**Background:**

The coronavirus disease-2019 (COVID-19) pandemic has caused myriad health, social, and economic stressors. To date, however, no known study has examined changes in mental health during the pandemic in the U.S. military veteran population.

**Methods:**

Data were analyzed from the 2019–2020 National Health and Resilience in Veterans Study, a nationally representative, prospective cohort survey of 3078 veterans. Pre-to-peri-pandemic changes in psychiatric symptoms were evaluated, as well as pre-pandemic risk and protective factors and pandemic-related correlates of increased psychiatric distress.

**Results:**

The prevalence of generalized anxiety disorder (GAD) positive screens increased from pre- to peri-pandemic (7.1% to 9.4%; *p* < 0.001) and was driven by an increase among veterans aged 45–64 years (8.2% to 13.5%; *p* < 0.001), but the prevalence of major depressive disorder and posttraumatic stress disorder positive screens remained stable. Using a continuous measure of psychiatric distress, an estimated 13.2% of veterans reported a clinically meaningful pre-to-peri-pandemic increase in distress (mean = 1.1 standard deviation). Veterans with a larger pre-pandemic social network size and secure attachment style were less likely to experience increased distress, whereas veterans reporting more pre-pandemic loneliness were more likely to experience increased distress. Concerns about pandemic-related social losses, mental health COVID-19 effects, and housing stability during the pandemic were associated with increased distress, over-and-above pre-pandemic factors.

**Conclusions:**

Although most U.S. veterans showed resilience to mental health problems nearly 1 year into the pandemic, the prevalence of GAD positive screens increased, particularly among middle-aged veterans, and one of seven veterans experienced increased distress. Clinical implications of these findings are discussed.

## Introduction

The coronavirus disease-2019 (COVID-19) pandemic has caused widespread social, health, and economic challenges. Pandemic-related stressors including risk of virus exposure which could lead to severe illness or death, social distancing, and lockdown measures may negatively impact mental health, which has led to calls for research on the population-level mental health effects of COVID-19 (Holmes et al., 2020). These impacts might be especially pronounced in the United States, which has suffered the highest number of confirmed COVID-19 cases and deaths in the world (Dong, Du, & Gardner, 2020), as well as an economic recession that has persisted into 2021. Indeed, repeated cross-sectional surveys of nationally representative samples of American adults have found high prevalence of psychiatric distress during the pandemic – with roughly a quarter of respondents (*v.* 6.5–8.1% in 2019) reporting symptoms of anxiety and depression in one survey (Czeisler et al., 2020) and 13.6% (*v.* 3.9% in 2018) reporting serious psychological distress in another (McGinty, Presskreischer, Han, & Barry, 2020b) – raising concerns that the prevalence of psychiatric disorders such as major depressive disorder (MDD), generalized anxiety disorder (GAD), and posttraumatic stress disorder (PTSD) may have increased.

Despite these findings, only a few longitudinal studies to date have examined changes in psychiatric distress in the U.S. population during the pandemic (Breslau et al., 2021; Daly & Robinson, 2020; McGinty, Presskreischer, Anderson, Han, & Barry, 2020a), and several of these studies did not assess pre-pandemic distress (Daly & Robinson, 2020; McGinty et al., 2020a). This is an important gap, as repeated cross-sectional surveys rely on measurements from different samples, often using different methodologies. Longitudinal assessments of the same cohort over time are needed to understand whether mental health has worsened for individuals relative to their own pre-pandemic mental health. To our knowledge, only one previous longitudinal study has measured pre-to-peri-pandemic change in psychiatric distress in a nationally representative sample of U.S. adults (Breslau et al., 2021). This study found that a similar proportion of the sample reported past-month serious psychological distress on the Kessler-6 (Kessler et al., 2003) in May 2020 as reported past-year serious distress in February 2019 (10.9% *v.* 10.2%). Although past-month estimates were not directly compared, the authors interpreted this similarity as an increase in distress based on a previous finding from a nationally representative study that the past-year estimate of serious distress was approximately double the past-month estimate (Hedden et al., 2012). Additional methodologically rigorous longitudinal studies of Americans, particularly in vulnerable subpopulations (Holmes et al., 2020), are needed to better understand the mental health burden of the COVID-19 public health crisis. In addition, information about factors associated with pandemic-related psychiatric distress in these vulnerable groups is essential to identifying individuals most in need of support, and to inform targeted clinical and policy interventions.

One subpopulation of the United States that may be uniquely vulnerable to psychiatric distress during the pandemic is military veterans. Veterans have greater exposure to childhood adversity and combat trauma (Katon et al., 2015), which are known to sensitize individuals to the negative effects of later stressors (Bandoli et al., 2017; Nichter, Hill, Norman, Haller, & Pietrzak, 2020a; Smid et al., 2015). In addition, veterans are more likely to have a history of psychiatric conditions, such as PTSD, suicidal behavior, and substance use disorders relative to the non-veteran population (Hoggatt, Lehavot, Krenek, Schweizer, & Simpson, 2017; Lehavot, Katon, Chen, Fortney, & Simpson, 2018; United States Department of Veterans Affairs Office of Mental Health & Suicide Prevention, 2019), which may be exacerbated by pandemic stressors (Murphy, Williamson, Baumann, Busuttil, & Fear, 2020). Indeed, one previous study of veterans with pre-existing psychiatric disorders found that pre-pandemic psychiatric symptom severity predicted suicide ideation during the pandemic (Na et al., 2021). Finally, relative to civilians, veterans have higher rates of medical morbidities such as cardiovascular disease, obesity, and diabetes (Hoerster et al., 2012; Lehavot, Hoerster, Nelson, Jakupcak, & Simpson, 2012), which may increase vulnerability to health-related stressors including risk of severe disease from COVID-19 infection and pandemic-related delays in obtaining routine health care (Földi et al., 2020; Ssentongo, Ssentongo, Heilbrunn, Ba, & Chinchilli, 2020). Despite these characteristics no known study has examined pandemic-related changes in the mental health of the general U.S. veteran population.

To address this gap, we analyzed data from a nationally representative sample of U.S. military veterans who completed surveys before and nearly 1 year into the pandemic. To identify changes in specific disorders, we first measured the pre- and peri-pandemic prevalence of positive screens for common psychiatric conditions; then, following prior research (Breslau et al., 2021), we used a continuous, transdiagnostic measure of psychiatric distress to assess variability in distress more sensitively and identify correlates of increased distress. We had three aims: (1) to examine changes in the prevalence of positive screens for MDD, GAD, and PTSD from pre-to-peri-pandemic; (2) to identify pre-pandemic risk and protective factors that predicted a clinically meaningful increase [i.e. 0.5 standard deviation (s.d.) or higher; Norman, Sloan, and Wyrwich, 2003; Ready et al., 2008; Schnurr et al., 2003] in psychiatric distress (i.e. continuously assessed composite score of MDD, GAD, and PTSD symptoms); and (3) to evaluate associations between COVID-19-related infection stressors, general pandemic stressors, and pandemic-related changes in risk factors, and increased psychiatric distress.

With respect to aim 1, we hypothesized small but significant pre-to-peri-pandemic increases in positive screens for psychiatric disorders, based on general population findings (Breslau et al., 2021) and greater pre-pandemic mental health problems among veterans (Hoglund & Schwartz, 2014). With respect to aim 2, we hypothesized that pre-pandemic factors that robustly predict psychiatric distress, such as prior trauma exposure, psychiatric and substance use problems, and loneliness (Bandoli et al., 2017; Kuwert, Knaevelsrud, & Pietrzak, 2014; Seal et al., 2011; Smid et al., 2015), would positively predict increased distress during the pandemic, whereas psychological and social characteristics previously shown to protect mental health would negatively predict distress (Pietrzak & Cook, 2013). With respect to aim 3, we hypothesized that COVID-19 infection stressors, general pandemic stressors, and increases in pre-pandemic risk factors would be associated with increased psychiatric distress (Birditt, Turkelson, Fingerman, Polenick, & Oya, 2021; Holingue et al., 2020; Holman, Thompson, Garfin, & Silver, 2020; McGinty et al., 2020a; Shi et al., 2020; Wang, Kala, & Jafar, 2020; Zheng et al., 2021).

## Methods

### Participants

Data were analyzed from two waves (hereafter: pre-pandemic and peri-pandemic) of the 2019–2020 National Health and Resilience in Veterans Study (NHRVS), which surveyed a nationally representative sample of 4069 U.S. military veterans. Pre-pandemic data collection began before the first documented case of COVID-19 infection in China and ended before implementation of the first COVID-19-related lockdown in the USA (median completion date: 21 November 2019). Peri-pandemic data were collected from 3078 veterans (75.6% of the original sample) approximately 1 year later, during the 2020 fall and winter surge of COVID-19 cases (median completion date: 14 November 2020). Recruitment and sample details of the 2019–2020 NHRVS have been reported previously (Hill, Nichter, Loflin, Norman, & Pietrzak, 2021). Briefly, the sample was drawn from KnowledgePanel^®^, a survey research panel of more than 50 000 households maintained by Ipsos. Ipsos statisticians computed post-stratification weights to permit generalizability of results to the U.S. veteran population using benchmark distributions from the Veterans Supplement of the most contemporaneous U.S. Census Current Population Survey (Bureau of the Census, 2019). All participants provided informed consent. The study was approved by the Human Subjects Committee of the VA Connecticut Healthcare System and carried out in accordance with the Declaration of Helsinki.

The sample consisted of veterans aged 22–99 years (mean age = 63.2; s.d. = 14.7). The sample was mostly male (91.6%); 79.3% were non-Hispanic Caucasian, 10.3% non-Hispanic African American, 6.0% Hispanic, and 4.4% mixed/other race. The sample included veterans who had served in all branches of the U.S. military [majority Army (47.3%), Navy (20.8%), or Air Force (18.9%)], 35.0% were combat veterans, 79.6% reported having enlisted in the military, and 20.1% reported utilizing VA as their primary source of healthcare.

### Assessments

At the pre-pandemic assessment, sociodemographic and military characteristics, psychosocial risk and protective factors, and current psychiatric conditions were assessed. At the peri-pandemic assessment, psychosocial factors and psychiatric conditions were reassessed using the same measures, and COVID-19 infection stressors (related to actual infection of the self or close others) and pandemic stressors (related to general pandemic stressors such as risk of disease, social distancing, and lockdowns) were assessed. Table 1 presents detailed descriptions of all the measures used to assess mental health outcomes, as well as all predictors and correlates that were examined in relation to changes in psychiatric distress.

**Table 1.** Measures of psychiatric, sociodemographic, military, and psychosocial variables and COVID-19 infection and pandemic stressors

Psychiatric conditions	
MDD	Score ⩾3 on the two depression items of the Patient Health Questionnaire for Depression and Anxiety (PHQ-4; Kroenke, Spitzer, Williams, & Löwe, 2009) in the past 2 weeks
GAD	Score ⩾3 on the two anxiety items of the PHQ-4 (Kroenke et al., 2009) in the past 2 weeks
PTSD	Past-month total score ⩾33 on PTSD Checklist for DSM-5 (PCL-5; Weathers *et al*., 2013b); veterans reported on past-month PTSD symptoms in relation to their self-reported ‘worst’ Criterion A trauma on the Life Events Checklist for DSM-5 (Weathers et al., 2013a)
Sociodemographic characteristics	Age (continuous), sex (male, female), race/ethnicity (white, non-white), education (college graduate or higher, up to high school diploma), marital status (married/living with partner, not), household income ($60 000 or more, less than $60 000), retirement status (retired, not)
Military characteristics	Combat veteran status (combat exposure, not), primary source of healthcare (VA, other), enlistment status (enlisted into military, drafted into military, commissioned into military)
Pre-pandemic psychosocial risk factors	
Adverse childhood experiences	Adverse Childhood Experiences Questionnaire score (ACEQ; Finkelhor, Shattuck, Turner, & Hamby, 2015)
Total traumas	Items endorsed on Life Events Checklist for DSM-5 (LEC-5; Weathers et al., 2013a)
Lifetime MDD or PTSD	Lifetime MDD was assessed according to DSM-5 diagnostic criteria using the Mini International Neuropsychiatric Interview (MINI; Sheehan, 2016). Lifetime PTSD was defined as a score of 33+ (Bovin et al., 2016) on the PCL-5 (Weathers et al., 2013b), which was modified to include lifetime ratings of all PTSD symptoms in relation to veterans' self-reported ‘worst’ Criterion A trauma on the LEC-5 (Weathers et al., 2013a). Veterans who met criteria for either disorder were coded positive for lifetime MDD or PTSD
Lifetime AUD or DUD	Lifetime AUD and DUD were defined as meeting DSM-5 diagnostic criteria for AUD or DUD, respectively, as assessed using the MINI (Sheehan, 2016). Veterans who met criteria for either disorder were coded positive for lifetime AUD or DUD
Past-year alcohol use problem severity	Alcohol Use Disorders Identification Test (AUDIT) total score (Saunders, Aasland, Babor, De La Fuente, & Grant, 1993)
Past-year days of non-prescription drug use	Number of days reported in response to the following question from the Screen of Drug Use (Tiet et al., 2015): How many days in the past year have you used *non-prescription* drugs?
Loneliness	Score on 3-item measure adapted from the UCLA Loneliness Scale (Hughes et al., 2004)
Pre-pandemic psychosocial protective factors	
Protective psychosocial characteristics	Factor score using the following six indicators: score on Purpose in Life Test-Short Form (Schulenberg, Schnetzer, and Buchanan, 2011); score on Connor–Davidson Resilience Scale-10 (Campbell-Sills & Stein, 2007); rating (1 = strongly disagree to 7 = strongly agree) on single-item measure of optimism from Life Orientation Test-Revised (‘In uncertain times, I usually expect the best’) (Scheier, Carver, & Bridges, 1994); rating (1 = strongly disagree to 7 = strongly agree) on single-item measure of gratitude from Gratitude Questionnaire (‘I have so much in life to be thankful for’) (McCullough, Emmons, & Tsang, 2002); rating (1 = strongly disagree to 7 = strongly agree) on single-item measure of curiosity/exploration from Curiosity and Exploration Inventory-II (‘I frequently find myself looking for new opportunities to grow as a person’) (Kashdan et al., 2009); rating (1 = strongly disagree to 7 = strongly agree) on single item measuring perceived level of community integration (‘I feel well integrated in my community’)
Social connectedness	Factor score using the following three measures as indicators: score on 5-item Medical Outcomes Study Social Support Scale (Sherbourne & Stewart, 1991); size of social network as assessed by the question, ‘About how many close friends and relatives do you have (people you feel at ease with and can talk to about what is on your mind)?’; secure attachment style as measured by the Attachment Style Questionnaire (ASQ; Hazan & Shaver, 1987)
Change in psychosocial risk factors from pre- to peri-pandemic	Increase in alcohol consumption (AUDIT consumption; Saunders et al., 1993), increase in alcohol use problem severity (AUDIT consequences; Saunders et al., 1993), increase in days of non-prescription drug use (Screen of Drug Use; Tiet et al., 2015), change in loneliness (UCLA Loneliness Scale; Hughes et al., 2004)
COVID-19 infection stressors	COVID-19 infection status (endorsement of: self-infected, know someone in household who was infected, know someone not in household who was infected, and know someone who died of COVID-19)
COVID-19 pandemic stressors	Questions from the Coronavirus Health Impact Survey (National Institute of Mental Health, 2020) were used to assess COVID-19-associated worries and concerns at the peri-pandemic assessment. Factor analysis revealed that these items loaded on five factors (eigen values = 1.01–4.94): COVID-19-related disease worries (e.g. ‘In the past month, how worried have you been about being infected with coronavirus?’); COVID-19 social restriction stress (e.g. ‘How stressful have these changes in social contacts been for you?’); COVID-19-associated socioeconomic stress (e.g. ‘In the past month, to what degree have changes associated to the pandemic created financial problems for you or your family?’); COVID-19-associated relationship difficulties (e.g. ‘Has the quality of the relationships between you and members of your family changed?’); and COVID-19-associated social engagement (e.g. ‘In the past month, how many people, from outside of your household, have you had an in-person conversation with?’)

COVID-19, coronavirus disease-2019; VA, veterans affairs; DSM-5, Diagnostic and Statistical Manual of Mental Disorders, 5th edition; MDD, major depressive disorder; GAD, generalized anxiety disorder; PTSD, posttraumatic stress disorder; AUD, alcohol use disorder; DUD, drug use disorder; UCLA, University of California, Los Angeles.

### Data analysis

Statistical analyses were performed using SPSS version 25 and R statistical software. Raw unweighted frequencies are reported. Missing data (<3%) were multiply imputed using chained equations. When computing prevalence and inferential statistics, post-stratification sampling weights were applied to allow for generalizability to the entire population of U.S. military veterans (Ipsos, 2020).

Data analyses proceeded in six stages. First, descriptive statistics were computed to estimate the prevalence of positive screens for MDD, GAD, and PTSD at pre-pandemic and 1-year peri-pandemic, and McNemar's tests were computed to evaluate differences between prevalence estimates at each wave. Second, using standardized *z* scores of a composite measure of psychiatric distress (i.e. increased MDD, GAD, or PTSD symptoms), change in distress from the pre- to peri-pandemic assessment was computed (*z* score_peri-pandemic_ − *z* score_pre-pandemic_), and then the sample was divided into veterans who did and did not experience a clinically meaningful increase in distress over this period using a minimum increase in distress of 0.5 s.d.. This threshold was selected based on evidence that a change of at least 0.5 s.d. represents a minimally clinically important difference for a broad range of measures, including psychiatric symptoms (Norman et al., 2003; Ready et al., 2008; Schnurr et al., 2003). To further validate this threshold in the current sample, rates of mental health treatment initiation during the pandemic were compared between veterans with and without increased psychiatric distress. Third, independent samples’ *t* tests for continuous variables and chi-squares for categorical variables were conducted to compare veterans with and without increased psychiatric distress with respect to sociodemographic and military characteristics, psychosocial risk and protective factors, pre-to-peri-pandemic changes in psychosocial risk factors, and COVID-19 infection and pandemic stressors. Fourth, a hierarchical logistic regression analysis was conducted to examine pre-pandemic predictors and peri-pandemic correlates of increased psychiatric distress. Variables that were statistically significantly associated with increased distress in bivariate analyses (*p* < 0.05) were entered as independent variables in this analysis. Step 1 included pre-pandemic sociodemographic, military, and psychosocial risk factors; step 2 included pre-pandemic psychosocial protective factors; and step 3 included pre-to-peri-pandemic changes in risk factors and COVID-19 infection and pandemic stressors. Fifth, a series of planned post-hoc analyses were conducted to identify individual variables comprising pre- (e.g. social connectedness) and peri- (e.g. COVID-19-related disease worries) pandemic composite measures independently associated with increased psychiatric distress. Sixth, a relative importance analysis (Tonidandel & LeBreton, 2011) was conducted using the R statistical package relaimpo (R Core Team, 2019) to determine the relative contribution of each significant variable identified in the hierarchical regression model and planned post-hoc analyses. This analysis partitioned the explained variance in increased psychiatric distress that was attributable to each significant independent variable while accounting for intercorrelations among these variables.

## Results

Table 2 presents the pre- and peri-pandemic prevalence estimates for MDD, GAD, and PTSD positive screens in the full sample and by age group. There was a statistically significant increase in the estimated prevalence of GAD from pre- to peri-pandemic in the full sample (7.1% to 9.4%), which was driven by an increase in GAD positive screens in veterans aged 45–64 years (8.2% to 13.5%).

**Table 2.** Pre-pandemic to 1-year peri-pandemic prevalence of positive screens for MDD, GAD, and PTSD among U.S. military veterans

	Pre-pandemic *N* (weighted %)	1-Year peri-pandemic *N* (weighted %)	Test of difference	*p*
MDD	198 (7.6)	225 (8.6)	3.31	0.07
Age 18–44	31 (18.8)	33 (21.6)	1.41	0.24
Age 45–64	97 (9.7)	102 (10.5)	0.57	0.45
Age 65+	197 (3.4)	219 (4.0)	1.16	0.28
GAD	161 (7.1)	241 (9.4)	18.92	**<0.001**
Age 18–44	35 (21.7)	39 (23.0)	0.28	0.60
Age 45–64	76 (8.2)	123 (13.5)	22.26	**<0.001**
Age 65+	49 (2.5)	74 (3.3)	1.41	0.24
PTSD	140 (4.8)	113 (4.1)	2.16	0.14
Age 18–44	12 (16.6)	16 (13.3)	1.16	0.28
Age 45–64	59 (13.3)	64 (11.8)	0.65	0.42
Age 65+	31 (3.6)	30 (3.5)	0.00	1.00

MDD, major depressive disorder; GAD, generalized anxiety disorder; PTSD, posttraumatic stress disorder.

Statistically significant *p* values (*p* < 0.05) are bolded.

A total of 389 (weighted 13.2%) veterans scored 0.5 s.d. or higher on a composite measure of change in psychiatric distress at the 1-year peri-pandemic assessment (mean change in distress = 1.1 s.d., s.d. = 0.6). Supporting the clinical validity of the 0.5 s.d. cut-off, veterans who met or exceeded this threshold were significantly more likely than those who did not to initiate mental health treatment during the pandemic (8.3% *v.* 3.4%, χ^2^ = 17.29, *p* < 0.001), and nearly 60% of these veterans screened positive for peri-pandemic MDD, GAD, and/or PTSD

Table 3 presents bivariate comparisons of veterans with and without increased psychiatric distress on measures of sociodemographic and military characteristics, pre-pandemic psychosocial risk and protective factors, pre-to-peri-pandemic changes in risk factors, and COVID-19-related infection and pandemic stressors. Compared with veterans who did not experience an increase in distress (mean change in distress = −0.20, s.d. = 0.5), veterans who did were younger and more likely to have enlisted into the military. They were also more likely to report several pre-pandemic risk factors, including more adverse childhood experiences, lifetime diagnosis of MDD and/or PTSD, lifetime diagnosis of alcohol use disorder and/or drug use disorder, and greater severity of past-year alcohol problems, frequency of past-year non-prescription drug use, and loneliness. They also scored lower on pre-pandemic measures of protective psychosocial characteristics and social connectedness and were more likely to report increases in loneliness and alcohol-related problems during the pandemic. In addition, they were more likely to endorse exposure to a range of infection and pandemic stressors, including knowing someone infected with COVID-19 and experiencing COVID-19-related disease worries, social restriction stress, socioeconomic stress, and relationship difficulties.

**Table 3.** Characteristics of U.S. military veterans with and without increased psychological distress from pre- to peri-pandemic

	No increase in psychological distress composite score *N* = 2672 (86.8%)	Increase in psychological distress composite score *N* = 389 (13.2%)		
	Mean (s.d.) or *N* (weighted %)	Mean (s.d.) or *N* (weighted %)	Test of difference	*p*
Sociodemographic characteristics				
Age	63.6 (14.7)	61.5 (14.4)	2.59	**0.010**
Male sex	2391 (91.9)	327 (89.3)	3.01	0.08
White race/ethnicity	2210 (79.2)	317 (79.1)	0.00	0.96
College graduate or higher education	1225 (34.5)	176 (32.3)	0.71	0.40
Married/partnered	1931 (73.8)	280 (75.3)	0.41	0.52
Household income $60k or higher	1620 (61.1)	224 (59.3)	0.48	0.49
Retired	1518 (47.2)	209 (44.5)	0.98	0.32
Military characteristics				
Combat veteran	916 (35.0)	130 (37.8)	1.17	0.28
VA primary source of healthcare	500 (20.3)	77 (19.8)	0.05	0.82
Enlisted into military	2004 (77.3)	316 (83.7)	8.26	**0.016**
Pre-pandemic psychosocial risk factors				
Baseline psychological distress	0.0 (0.6)	0.0 (0.9)	0.17	0.86
Adverse childhood experiences	1.4 (1.9)	1.7 (2.0)	−3.07	**0.002**
Total traumas	8.9 (8.3)	9.4 (8.2)	−1.11	0.27
Lifetime MDD and/or PTSD	466 (20.2)	123 (34.6)	38.89	**<0.001**
Lifetime AUD and/or DUD	1065 (41.8)	197 (50.5)	10.26	**0.001**
Past-year alcohol use problem severity	3.0 (4.4)	3.9 (4.7)	−3.48	**0.001**
Past-year days of non-prescription drug use	12.8 (60.4)	22.2 (79.5)	−2.18	**0.030**
Loneliness	4.4 (1.8)	5.3 (1.9)	−9.33	**<0.001**
Pre-pandemic psychosocial protective factors				
Protective psychosocial characteristics	0.1 (1.0)	−0.3 (0.9)	8.90	**<0.001**
Social connectedness	0.1 (1.0)	−0.4 (0.9)	8.34	**<0.001**
Change in risk factors from pre- to peri-pandemic				
Increase in alcohol consumption	451 (17.7)	72 (19.1)	0.45	0.50
Increase in alcohol-related problems	522 (7.7)	99 (14.9)	22.14	**<0.001**
Increase in non-prescription drug use	52 (2.2)	14 (3.5)	2.21	0.14
Change in loneliness	−0.1 (1.3)	0.2 (1.5)	−4.12	**<0.001**
COVID-19 infection stressors				
Infected with COVID-19	198 (8.0)	34 (9.8)	1.46	0.23
Someone in household infected with COVID-19	169 (7.4)	27 (8.3)	0.36	0.55
Know someone infected with COVID-19	198 (40.8)	34 (48.3)	7.82	**0.005**
Know someone who died of COVID-19	147 (5.5)	26 (5.6)	0.00	0.95
COVID-19 pandemic stressors				
COVID-19-related disease worries	−0.1 (1.0)	0.3 (1.0)	−6.94	**<0.001**
COVID-19-related social restriction stress	0.0 (1.0)	0.3 (1.0)	−5.64	**<0.001**
COVID-19-related socioeconomic stress	−0.1 (0.9)	0.3 (1.3)	−5.34	**<0.001**
COVID-19-related relationship difficulties	−0.1 (1.0)	0.3 (1.2)	−5.82	**<0.001**
Social engagement during pandemic	0.0 (1.0)	0.0 (1.1)	0.52	0.61
Hours of COVID-19-related media consumed per week	1.6 (2.1)	1.7 (2.1)	−1.11	0.27

s.d., standard deviation; VA, veterans affairs; MDD, major depressive disorder; PTSD, posttraumatic stress disorder; AUD, alcohol use disorder; DUD, drug use disorder; COVID-19, coronavirus disease 2019.

Increase in alcohol problems and increase in non-prescription drug use are binary variables; veterans with alcohol use problems or non-prescription drug use that increased by 0.5 s.d. or more from pre-pandemic to peri-pandemic were coded positive for respective increases in alcohol problems and drug use. Statistically significant *p* values (*p* < 0.05) are bolded.

Table 4 presents results of a hierarchical logistic regression analysis examining independent pre-pandemic predictors and COVID-19-related correlates of increased psychiatric distress. In step 1 of the model (pre-pandemic risk factors), fewer adverse childhood experiences and greater severity of past-year alcohol problems and loneliness predicted increased psychiatric distress. In step 2 of the model (pre-pandemic protective factors), veterans with higher levels of social connectedness were less likely to experience increased distress. In step 3 of the model (COVID-19-related changes and stressors), veterans with greater increases in loneliness and higher levels of COVID-19-related disease worries, social restriction and socioeconomic stress, and relationship difficulties were significantly more likely to experience increased psychiatric distress.

**Table 4.** Results of hierarchical logistic regression analysis of predictors and correlates of increased psychological distress

	*B*	s.e.	Wald	*p*	AOR (95% CI)
Step 1: Pre-pandemic risk factors					
Age	0.00	0.00	0.10	0.76	1.00 (0.99–1.01)
Enlistment status					
Enlisted	–	–	–	–	1 (reference)
Drafted	−0.37	0.23	2.57	0.11	0.69 (0.44–1.08)
Commissioned	−0.16	0.22	0.50	0.48	0.86 (0.56–1.32)
Adverse childhood experiences	−0.09	0.03	6.96	**0.008**	**0.91 (0.85–0.98)**
Lifetime MDD and/or PTSD	−0.02	0.16	0.02	0.87	0.97 (0.71–1.34)
Lifetime AUD and/or DUD	−0.11	0.13	0.67	0.41	0.90 (0.69–1.17)
Alcohol use problem severity	0.03	0.01	6.03	**0.014**	**1.03 (1.01–1.06)**
Past-year non-prescription drug use	0.00	0.00	1.43	0.23	1.00 (1.00–1.00)
Loneliness	0.17	0.05	11.67	**0.001**	**1.18 (1.07–1.30)**
*R^2^: 0.06*					
Step 2: Pre-pandemic protective factors					
Protective psychosocial characteristics	−0.11	0.08	2.25	0.13	0.89 (0.77–1.04)
Social connectedness	−0.21	0.09	5.65	**0.017**	**0.81 (0.69–0.96)**
*R^2^: 0.07*					
*R^2^ change: 0.01*					
Step 3: COVID-19-related changes and stressors					
Increase in alcohol problems	0.24	0.14	2.76	0.10	1.27 (0.96–1.68)
Change in loneliness	0.25	0.05	26.37	**<0.001**	**1.28 (1.16–1.40)**
Know someone infected with COVID-19	0.03	0.13	0.07	0.80	1.03 (0.81–1.32)
COVID-19-related disease worries	0.37	0.06	35.66	**<0.001**	**1.44 (1.28–1.62)**
COVID-19-related social restriction stress	0.22	0.06	13.94	**<0.001**	**1.25 (1.11–1.40)**
COVID-19-related socioeconomic stress	0.19	0.05	13.00	**<0.001**	**1.21 (1.09–1.34)**
COVID-19-related relationship difficulties	0.22	0.06	14.97	**<0.001**	**1.25 (1.12–1.40)**
*R^2^: 0.16*					
*R^2^ change: 0.09*					

AOR, adjusted odds ratio; CI, confidence interval; MDD, major depressive disorder; PTSD, posttraumatic stress disorder; AUD, alcohol use disorder; DUD, drug use disorder; COVID-19, coronavirus disease-2019.

Statistically significant *p* values (*p* < 0.05) are bolded.

Results of planned post-hoc analyses revealed that veterans with a secure attachment style [Wald χ^2^ = 8.02, *p* = 0.005; odds ratio (OR) 0.65, 95% confidence interval (CI) 0.48–0.87] and larger social network (Wald χ^2^ = 3.99, *p* = 0.046; OR 0.98, 95% CI 0.96–0.99) pre-pandemic were significantly less likely to experience increased psychiatric distress 1 year into the pandemic. With regard to pandemic-related items, increased psychiatric distress was associated with greater concern about the stability of one's living situation (Wald χ^2^ = 36.64, *p* < 0.001; OR 1.67, 95% CI 1.42–1.98), worries about COVID-19 affecting one's mental health (Wald χ^2^ = 11.90, *p* = 0.001; OR 1.24, 95% CI 1.10–1.40), stress related to changes in social contacts (Wald χ^2^ = 11.22, *p* = 0.001; OR 1.31, 95% CI 1.12–1.53), worsening quality of one's relationships with friends (Wald χ^2^ = 6.68, *p* = 0.010; OR 1.30, 95% CI 1.06–1.58), and worries about COVID-19 infecting friends or family (Wald χ^2^ = 6.49, *p* = 0.011; OR 1.18, 95% CI 1.04–1.35).

**Fig. 1.**
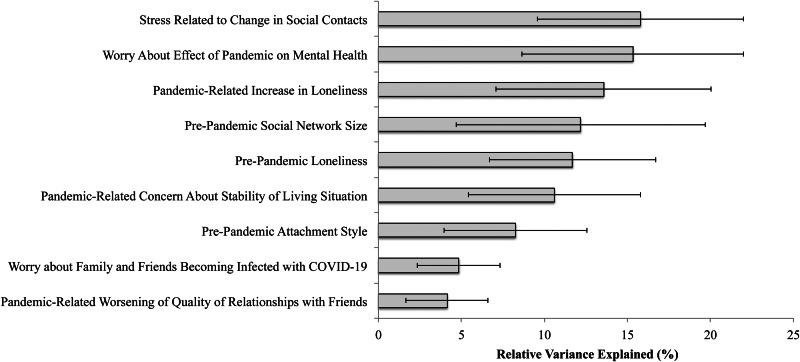
Results of relative importance analysis of significant correlates of increased psychological distress from pre- to peri-pandemic. *Note.* COVID-19=coronavirus disease 2019; Error bars represent 95% confidence intervals. Adverse childhood experiences (0.8% relative variance explained) and pre-pandemic alcohol use severity (2.8% relative variance explained) were not significant predictors of increased psychiatric distress in the relative importance analysis (both p's>0.05).

As shown in Fig. 1, a relative importance analysis revealed that social network size [12.2% relative variance explained (RVE)] and loneliness (11.7% RVE) were the strongest pre-pandemic predictors of increased psychiatric distress. Stress related to changes in social contacts during the pandemic (15.8% RVE), worry about effects of the pandemic on one's mental health (15.3% RVE), and increased loneliness during the pandemic (13.6% RVE) were the strongest peri-pandemic correlates of this outcome.

## Discussion

To our knowledge, this is the first study to examine pandemic-related mental health changes in a nationally representative sample of U.S. military veterans. The current findings suggest that veterans evidenced considerable mental health resilience during the COVID-19 pandemic, but with substantial heterogeneity in individual vulnerability. Consistent with our first hypothesis, we found a pre-to-peri-pandemic increase in positive screens for GAD. This increase translates into substantial negative impact when extrapolated to the national population of nearly 18 million veterans (Vespa, 2020); using estimated population benchmarks (Bureau of the Census, 2019), more than 400 000 additional veterans may have screened positive for GAD 1-year into the pandemic. Elevated GAD was driven largely by veterans aged 45–64 years, among whom GAD positive screens increased by more than 65% from pre-to-peri pandemic (8.2% to 13.5%). This result was somewhat surprising given previous findings of greater pandemic-related distress among young adults (Breslau et al., 2021; Czeisler et al., 2020; Holman et al., 2020; McGinty et al., 2020b). However, data from previous studies came from early in the pandemic, and there is some evidence that age differences in psychiatric distress decreased over time (Banks, Fancourt, & Xu, 2021). Our results suggest that middle-aged veterans may have experienced unique challenges as the pandemic progressed, such as overlapping stressors related to the health and economic impacts of the pandemic. Indeed, post-hoc comparisons of the three age groups revealed that middle-aged veterans reported higher levels of COVID-19-related disease worries than younger veterans, and greater COVID-19-related socioeconomic stress than older veterans. As middle-aged individuals typically are not yet retired but are also of an age associated with more severe health impacts of COVID-19 (Fang et al., 2020), this group may have found it difficult to continuously self-isolate due to work-related responsibilities, and stressful to attend in-person work due to heightened disease risk. Moreover, this age cohort often balances work and care-taking responsibilities (Hopps, Iadeluca, McDonald, & Makinson, 2017), and they may have been disproportionately impacted by persistent school closures and remote learning (Garbe, Ogurlu, Logan, & Cook, 2020).

Although this increased anxiety is consistent with elevated distress observed in cross-sectional surveys (Czeisler et al., 2020; Holman et al., 2020; McGinty et al., 2020b), the increase was smaller in the current study, and we observed notable stability in the prevalence of MDD and PTSD positive screens. This may be because the current study assessed the same cohort pre- and peri-pandemic, which is essential to accurately assessing pandemic-related changes over time. Indeed, our findings are consistent with a developing literature of longitudinal studies showing only small within-subject, population-level changes in mental health outcomes during the COVID-19 pandemic. For example, a recent rapid-review and meta-analysis of 25 longitudinal and natural experiment studies consisting of more than 70 000 participants from around the globe found small effects of COVID-19 lockdowns on anxiety (*g* = 0.17) and depression (*g* = 0.15), and null effects on general distress, positive functioning, and suicide risk (Prati & Mancini, 2021). Similarly, roughly 75% of respondents in the only previous U.S. population-based longitudinal study reported low psychiatric distress at both pre- and peri-pandemic assessments (Breslau et al., 2021). The current study extends this literature by demonstrating a similar pattern among U.S. military veterans, a population that has been characterized as high-risk for mental health impacts of the COVID-19 pandemic (Amsalem et al., 2021).

Despite considerable resilience, however, pandemic-related mental health changes among veterans were heterogeneous, mirroring research showing wide individual variability in responses to acute stress (Bonanno & Mancini, 2012). In fact, when we examined within-person changes in transdiagnostic symptoms of MDD, GAD, and PTSD, nearly 1 in 7 veterans experienced more than a 0.5 s.d. increase in distress relative to their pre-pandemic levels. This estimated 13.2% of veterans with increased distress is strikingly similar to the 12.8% of U.S. adults who reported increased distress from pre- to peri-pandemic in a previous longitudinal study (Breslau et al., 2021). As this increase was not fully accounted for by the increased prevalence of positive GAD screens, it appears that a significant proportion of the distress that emerged during the pandemic was sub-diagnostic. However, the magnitude of this increase suggests a need for intervention to prevent increased distress from transitioning into more persistent psychiatric disorders. Thus, these findings highlight the importance of delineating risk factors for pandemic-related psychiatric distress to target interventions to vulnerable veterans.

Results of a relative importance analysis revealed that pre-pandemic social factors – specifically, social network size, loneliness, and attachment style – predicted increased psychiatric distress during the pandemic. Specifically, veterans with a larger pre-pandemic social network size and secure attachment style were less likely to experience an increase in distress, whereas veterans with greater pre-pandemic loneliness were more likely to experience such an increase. Collectively, pre-pandemic social factors accounted for roughly a third of the explained variance in increased distress. These findings, which are consistent with a literature showing that social support may help buffer the deleterious effects of stressors through psychobiological mechanisms (Ditzen & Heinrichs, 2014), align with calls for health care providers to regularly monitor social factors in populations at increased risk for pandemic-related distress (Killgore, Cloonan, Taylor, Lucas, & Dailey, 2020). Brief, validated measures such as the Three-Item Loneliness Scale (Hughes, Waite, Hawkley, & Cacioppo, 2004) could be used to screen veterans in primary and mental health care settings.

Results of a relative importance analysis also identified potentially modifiable pandemic-related stressors associated with increased psychiatric distress, including social losses – stress about changes in social contacts, increased loneliness, and worsening in the quality of friend relationships – worry about effects of the pandemic on mental health, and concern about housing stability during the pandemic. These findings support our third hypothesis and have several practical implications. First, associations of social loss with increased distress highlight the importance of prevention efforts such as outreach and messaging campaigns to share strategies for virtual social connection and to destigmatize loneliness. Second, associations of distress with worry about pandemic effects on mental health underscore the need for public investment to reduce barriers to mental health care. As telehealth delivery is as effective as in-person delivery of evidence-based anxiety, depression, and PTSD treatments (Morland et al., 2020; Tuerk, Keller, & Acierno, 2018), expansion of these services is warranted. Resources to provide smart tablets, as successfully piloted by the Department of Veterans Affairs (Zulman et al., 2019), could expand access to low-income households where smart phone and laptop ownership are not universal (Nadkarni et al., 2020). Brief video interventions that boost treatment-seeking intentions may also be helpful (Amsalem et al., 2021), as many veterans even with severe psychological symptoms are not engaged in treatment (Nichter, Hill, Norman, Haller, & Pietrzak, 2020b).

Third, associations of distress with housing stability concerns highlight the need for continued government financial support for vulnerable individuals – perhaps particularly for middle-aged veterans. The high levels of financial stress and disease worries reported by this age cohort suggest greater fears of job instability and virus exposure, which may happen in work settings. This distress may signal a need for extension of unemployment insurance and direct payments introduced early in the pandemic to offset the impact of persistent job losses and concerns about the safety of returning to work. To abate housing concerns, expansion of housing-specific interventions such as rental and mortgage assistance and eviction diversion programs may be beneficial (Benfer et al., 2021). For veterans specifically, this relief may come from the recent allocation of additional funding to the Housing and Urban Development-Veterans Affairs Supportive Housing program (HUD-VASH), which provides rental assistance and housing vouchers to veterans at risk of homelessness. Our findings support the expansion of this program's priorities during the pandemic to include a greater focus on homelessness prevention, which could include direct renter payments to veterans accumulating significant debt that may result in homelessness when eviction moratoria are lifted.

The current study has important strengths, including the use of a large, nationally representative sample of veterans, a pre-to-peri-pandemic longitudinal design, and assessment of the prevalence of psychiatric conditions in late 2020, after pandemic-related mental health effects had additional time to unfold. However, results must also be interpreted in the context of several limitations. First, the sample consisted of predominantly older, white, and male U.S. military veterans; distress might be higher among individuals underrepresented in the veteran population. As previous research has found greater pandemic-related distress among women (Breslau et al., 2021), ethnic/racial minorities (McGinty et al., 2020b), and young adults (Breslau et al., 2021; McGinty et al., 2020b), the current results may not generalize to civilians or veterans in these groups, or to veterans who are institutionalized or homeless. Second, although retention was relatively high (75.6%), veterans with a pre-pandemic diagnosis of MDD, GAD, and/or PTSD were less likely to complete the follow-up survey than veterans without these diagnoses (67.4% *v.* 76.7%). Therefore, it is possible that the prevalence of these disorders may have been underestimated due to retention or survivorship bias (Czeisler, Wiley, Czeisler, Rajaratnam, & Howard, 2021). Third, although we assessed peri-pandemic stress later in the pandemic than previous studies, mental health effects of the pandemic may be delayed and manifest over time, as economic, health, and social consequences of the pandemic compound. Fourth, screening instruments were used to assess psychiatric symptoms; studies using clinical interviews are needed to confirm the prevalence estimates reported in this study. In addition, we selected a 0.5 s.d. cut-off to indicate increased psychiatric distress, which, although identified in previous research as a valid indicator of clinical distress (e.g. Norman et al., 2003) and significantly associated with mental health treatment-seeking in the current sample, may or may not indicate a transition to a clinically concerning level of symptoms; indeed, some studies have used higher thresholds to define adverse mental health outcomes (e.g. top 10% of the distribution of change scores; Chen and Hardy, 2009). However, we note that the group created using this cut-off experienced, on average, greater than a standard deviation increase in distress (mean change in distress = 1.1 s.d., s.d. = 0.6); in fact, the prevalence of positive screens for MDD, GAD, and/or PTSD in this group increased from 9.4% at pre-pandemic to 54.2% at peri-pandemic. Although it is possible that some of the increased distress we observed, particularly lower magnitude increases, reflected normal fluctuations in symptoms, we posit that even sub-diagnostic increases in distress are important to identify as they may portend more substantial problems. Fifth, COVID-19-related correlates of increased psychiatric distress were measured simultaneously with peri-pandemic distress, making the temporal direction of these associations unclear; it is possible that increased mental health symptoms caused veterans to report greater perceived stress related to the pandemic.

## Conclusions

These limitations notwithstanding, the current study provides the first known nationally representative data on the mental health impact of the COVID-19 pandemic on U.S. military veterans. Our findings, which align with the broader literature showing high rates of adaptive functioning in the face of trauma, adversity, and disaster (Bonanno, 2005; Norris, Tracy, & Galea, 2009; North et al., 2002), reflect the remarkable human ability to adapt even to profound disruptions to daily life wrought by the pandemic. However, they also highlight disparities in the effects of the pandemic and the resultant need for targeted interventions to protect those at risk of deteriorating mental health, particularly middle-aged veterans and veterans with low social connectedness.

To better understand trajectories of risk and resilience, it will be important to continue tracking long-term changes in mental health, ideally using continuous measures of psychopathology, which are more sensitive to detecting within-person changes in distress. Because of the ongoing nature of the pandemic and the continued presence of imminent stressors, it is unclear whether the elevated distress we observed will result in durable and functionally impairing psychiatric disorders. Prior research has found that population-level increases in distress following natural disasters often return to pre-disaster levels over time (Pietrzak et al., 2012). Although still early, emerging evidence about the persistence of distress during the pandemic in the USA is mixed; one study found an early spike in distress in spring of 2020 that returned to baseline levels by summer (Daly & Robinson, 2020), but another study found consistent high distress in April and July (McGinty et al., 2020a). Because data from the current study were collected in the fall of 2020, the elevated distress we observed may be an indicator of more persistent mental health problems. Post-pandemic follow-up will be essential to understanding the prevalence and determinants of acute *v.* long-term changes in mental health and functioning in veterans and the population at large.
